# Sleep phase and pre-sleep arousal predicted co-developmental trajectories of pain and insomnia within adolescence

**DOI:** 10.1038/s41598-022-08207-y

**Published:** 2022-03-16

**Authors:** Tor Arnison, Martien G. S. Schrooten, Serena Bauducco, Markus Jansson-Fröjmark, Jonas Persson

**Affiliations:** 1 School of Law, Psychology and Social Work, Örebro University, Örebro, Sweden; 2 Centre for Psychiatry Research, Department of Clinical Neuroscience, Karolinska Institute, Stockholm, Sweden; 3 Stockholm Health Care Services, Region Stockholm, Stockholm, Sweden; 4 Aging Research Center (ARC), Karolinska Institute and Stockholm University, Stockholm, Sweden

**Keywords:** Psychiatric disorders, Public health, Epidemiology, Paediatric research, Preclinical research, Risk factors, Pain management, Pain, Comorbidities

## Abstract

The onset of both chronic pain and insomnia is high during adolescence. Although a bidirectional relationship between pain and insomnia has support, how pain and sleep co-develop throughout adolescence remains unknown. Sleep–wake patterns, pre-sleep behavior and pre-sleep arousal may influence the co-development of pain and insomnia. Four waves of longitudinal self-report data were used (N^baseline^ = 2767, Age^baseline^ M = 13.65 years, SD = 0.65). Multidimensional growth mixture modeling was used to identify four subgroups of adolescents with different concurrent trajectories of pain and insomnia. The trajectories followed each other across time in all classes: one class of consistently low pain and insomnia (68.7%), one class with persistent high symptoms (4.9%), as well as one class of increasing (13.9%), and one of decreasing (12.5%), trajectories. Later sleep–wake patterns and more pre-sleep cognitive-emotional arousal predicted both increasing and decreasing trajectories of concurrent pain and insomnia. The current study showed that developmental trajectories of pain and insomnia follow each other within adolescents and across adolescence. Both sleep-phase focused interventions as well as psychological interventions that focus on pre-sleep cognitive-emotional arousal may prove beneficial for adolescents with comorbid pain and insomnia.

## Introduction

Chronic pain is a leading cause of years lived with disability worldwide^1^. Insomnia appears to be a considerable driver of pain^2,3^, also during adolescence^4^, which has been identified as a critical period for their co-development^5^. The incidence and onset of both chronic pain^6^ and insomnia^7^ is high across adolescence. About 50% of adolescents who suffer from chronic pain also have insomnia^8,9^, and this comorbidity may be more disabling than each condition on its own^9^.

Evidence supporting an association between pain and insomnia continues to grow. Yet, little is known about how pain and insomnia co-develop over time. Which co-developmental trajectories of pain and insomnia can be distinguished, and what factors predict these different patterns? It is known that adolescents who develop chronic pain^10^ or insomnia^11^ are at high risk of maintaining these conditions into adulthood. A better understanding of how pain and insomnia co-develop throughout adolescence may not only reveal specific targets for intervention for adolescents with comorbid insomnia and chronic pain but is also needed to develop effective strategies to prevent the maintenance and exacerbation of chronic pain and sleep conditions.

We are unaware of studies that have specifically addressed individual differences in longitudinal co-development of insomnia and pain. A recent review established that a change towards poorer sleep can predict a future increase in pain, but it remains unclear whether an improvement in sleep quality can predict a decrease in pain^12^. Can these mixed findings on the reciprocal relationship between sleep disturbance and pain^2^ be attributed to (unobserved) subgroups in the general population of adolescents who show differentiated sleep-pain relationships? Are there subgroups of adolescents with only insomnia or pain problems? Such subgroups may explain heterogeneity of results in previous studies.

Relatively little is known of risk factors for concurrent insomnia and pain, but potential candidates include female gender, older age, depression and anxiety, short sleep duration, stress, low socioeconomic status (SES), and immigrant background^8,13,14^. Since insomnia has been found to be a stronger predictor of future pain than vice versa, a focus on sleep-related risk factors has been proposed when examining comorbid pain and insomnia^8^. Although such risk factors have the clearest association with insomnia, it is not known if they predict how both pain and insomnia co-develop over time.

The increasing prevalence of pain and insomnia during adolescence temporally coincides with both pubertal development and a pronounced delay in sleep–wake patterns^15^, the latter mainly driven by a reduced build-up of sleep pressure and a delayed circadian rhythm^5^. Therefore, the concurrent increases of pain and insomnia in adolescents have been explained in terms of developmental changes associated with puberty or by factors specifically related to the delay in sleep–wake patterns^5,15^, such as melatonin. For example, delayed dim light melatonin onset is associated with delayed sleep–wake patterns in adolescents^7,16^ as well as with pain^17^. Emerging evidence also suggests that both pain and sleep are regulated by circadian rhythm; the suprachiasmatic nucleus synchronizes the circadian clocks in peripheral tissues through secretion of, for example, dopamine and cytokines, which are each involved in the regulation of both pain and sleep^18^. In cross-sectional studies, a late sleep-phase has been associated with insomnia and depressed mood in adolescents^19,20^ and pain in adults^21,22^. However, no longitudinal studies to date have explored the effect of a late sleep phase on concurrent pain and insomnia.

The increase in pain and insomnia during adolescence may not only be due to physiological factors, since adolescents also show a distinct increase in maladaptive late-night behavior concurrently with the advancement in sleep phase^23^. Pre-sleep arousal and poor sleep hygiene habits have been shown to predict insomnia in adolescents with comorbid pain^9,24^. Moreover, cognitive and physiological hyperarousal has been found to be a main etiological factor in the pathogenesis of insomnia^7^, and has been associated both with pain generally^17^, as well as the interaction between sleep and pain specifically^5^. The question of whether and how these risk factors simultaneously influence pain, beyond the indirect effect through insomnia, remains unclear. As with a late sleep phase^25^, pre-sleep arousal predicts depressed mood^26^, which, in turn, is established as an important mediator in the insomnia-pain relationship^27^. Depressed mood may therefore be an underlying mechanism linking a late sleep phase and pre-sleep arousal to insomnia and pain.

The purpose of the current study was threefold: First, to identify subgroups of adolescents with unique co-developmental patterns of pain and insomnia by using a person-oriented multidimensional growth mixture modeling approach in a large longitudinal community sample of adolescents (N = 2767) spanning from 13/14 to 16/17 years old. Second, to describe the subgroups on demographic and mental health variables. Third, to explore the predictive effects of sleep phase and pre-sleep cognitive-emotional arousal (e.g., worry, rumination) or pre-sleep behavior (e.g., chatting with friends), on longitudinal growth trajectories of concurrent pain and insomnia. The resulting insights may prove useful in uniquely adapting interventions for adolescents with comorbid chronic pain and insomnia.

## Method

### Participants and procedure

This study is part of the Three Cities Study, a 5-year longitudinal study in Sweden that sought to better understand risk factors and mechanisms underlying mental health problems among adolescents. The program targeted adolescents in 7th and 8th grade (13 and 14 years old; N = 3 336) in 18 public upper secondary schools in three cities in central Sweden and data was acquired for four consecutive years after the baseline measurement (2014–2018). These participants filled out a battery of self-report questionnaires (paper-and-pencil format) each spring, in their classroom during school hours. Teachers left the classroom while trained test leaders supervised the procedure. The adolescents had 90 min to complete the questionnaires, including a short snack break. Each class received 300 SEK for participating. To reduce risks for sampling bias, the Three Cities Study used passive consent from the parents and active consent from adolescents^28^. The parents of 122 adolescents (3.7%) returned a prepaid letter where they declined participation, and, additionally, 447 adolescents (13.4%) either declined participation or were absent on the day of the first data collection. This resulted in a baseline sample of N = 2767 (82.9% of the N = 3336 target population: M_age_ = 13.65 (SD = 0.65). The analyses reported in this article included the first four data waves since data on the predictor variables were not collected in the fifth wave of data collection. All cases that had any data on the main pain and insomnia variables at baseline were included in the analyses. This resulted in an analytic sample of N = 2755 participants (82.6% of baseline target population, 99.6% of baseline sample). 1384 adolescents (50% of baseline sample) participated in all four measurement occasions, 2167 (78.3%) adolescents participated in at least three, 2619 (94.7%) in at least two, and 148 (5.3%) only participated at baseline. The study was approved by the regional Ethical Board in Uppsala (No 2013/384).

To investigate whether attrition across the four yearly measurement occasions (T1 to T4) was influenced by any systematic bias, we regressed attrition (number of missed measurement occasions, ranging from 1 to 3) on the baseline demographic characteristics of the adolescents (gender, age, SES and immigrant background) and all the other study variables (insomnia, sleep duration, sleep phase, depression, anxiety, pain frequency, pain intensity, pain interference, sleep-related behavior). Older adolescents (Wald χ^2^(1) = 28.57, *p* < 0.001), adolescents not born in Sweden or whose parents were not born in Sweden (Wald χ^2^(1) = 23.31, *p* < 0.001), or with a later sleep phase (Wald χ^2^(1) = 8.23, *p* = 0.004) were more likely to drop out from the study.

### Measures

#### Descriptive measures at baseline

Age, gender, SES, immigrant background, stress, depression, anxiety, sleep duration, insomnia, pain intensity, pain frequency, and pain interference were assessed at baseline (T1). *Age* was measured by asking the respondents to indicate their chronological age in years. *Gender* was measured by the respondent indicating “boy” or “girl” on one dichotomous item. *SES* was assessed using the four items of the *Family Affluence Scale, version 2 (FAS-II),* from WHO’s Health Behavior in School-aged Children (HBSC)—survey^29^. The respondent was instructed to indicate whether they had their own bedroom in the household, how many cars the household had, how many computers the household currently owned, and how many times the household went on a holiday during the last 12 months. The mean on FAS-II in the Swedish HBSC-survey (N = 23,088) was *M* = 6.28 (*SD* = 1.67). The cut-off for *low SES* was set at 4.61, one standard deviation below the population mean^29^. *Immigrant status* was assessed by the respondent indicating whether they were born in Sweden and where either of their parents were born (Sweden, Scandinavia, Europe or outside of Europe). Immigrant background was defined as being born in Sweden or having one parent being born in Sweden (i.e., non-immigrant background), or being born outside of Sweden or having both parents being born outside of Sweden (i.e. immigrant background), according to the official definition of Statistics Sweden^30^. *Depressive symptoms* were assessed with the Center for Epidemiology Studies Depression Scale for Children (CES-DC). CES-DC is a 20-item Likert scale with possible responses ranging from 0 (“not at all”) to 3 (“a lot”), resulting in a total score ranging from 0 to 60^31^ At T1, possible responses ranged from 1 to 5 and were adjusted to the original scaling using a POMP method^32^. CES-DC has shown good reliability and validity when applied on a sample of Swedish adolescents^31^, and the cut-off for clinical depression was set at a total score of at least 24^31^. In the current sample, the internal consistency of the scale at T1 was Cronbach’s α = 0.84. *Anxiety* was assessed with the Overall Anxiety Severity and Impairment Scale (OASIS). OASIS includes five Likert scale items assessing the degree of anxiety and impairment due to anxiety experienced during the last week. Possible responses range from 0 (“no”/”none”/”not at all”) to 4 (“constant”/”extreme”/”all the time”), resulting in a total score ranging from 0 to 20^33^. The cutoff for clinical anxiety problems is 8^33^, and the scale has previously shown good psychometric properties^33^. Cronbach’s alpha at T1 in the current study was α = 0.87. *Stress* was assessed with the Adolescent Stress Questionnaire, Short version^34^. Adolescents indicated how much stress from various social domains they have experienced in the past 6 months, on a 27 item, five-point Likert scale, ranging from 0 = not at all stressful to 4 = very stressful. The internal consistency at timepoint 1 was α = 0.87.

#### Pain measures

Pain intensity, pain frequency, and pain interference were measured separately for the three most common pain types in adolescents^6^: abdominal pain, headache, and musculoskeletal pain (i.e., pain in the neck, shoulders or back).

*Pain intensity* was measured by one item where the participants rated their average pain intensity during the last 6 months on a 10-point scale ranging from 0 (“not at all”) to 9 (“very painful”)^35^*.* In T3, the intensity measures for headache and abdominal pain asked for the average pain during *the last 2 months* instead for *the last 6 months*. To assess the potential impact of this, we fitted a longitudinal confirmatory factor analysis including the pain intensity measures for the three pain types across the four measurement occasions and tested for measurement invariance. The model met the assumption of strong measurement invariance, indicating that the meaning of the measures remained constant across the four measurement occasions, and that the deviation in T3 was inconsequential. For more details on these analyses, see the Supplementary Information.

*Pain frequency*; how often the participant had experienced pain, on average, during the last 6 months, was assessed with one item from the HBSC Symptom Checklist^36^, with the possible answers on a five-point Likert scale being “rarely or never” (0), “about every month” (1), “about every week” (2), “more than once per week” (3), and “about every day” (4).

*Pain interference*: how much the pain severely interfered with three life domains—school work, friend relationships and leisure activities. Possible responses were “no”, scored as 2, “yes, a bit”, scored as 1, and “yes, definitely”, scored as 0. The scores were reversed and summed across the three domains into a total score, ranging from 0 to 6. The measure was adapted from the Social Phobia Screening Questionnaire^37^ for the purpose of the Three Cities Study and has been used in previously published studies^4,38^.

*Pain grades*. A composite “pain grade” was constructed based on the above-mentioned facets of the pain experience (pain intensity, pain frequency, and pain interference)^14,38^, as described in Table 1. We calculated separate pain grades for musculoskeletal pain, abdominal pain, and headache. An average pain grade was calculated by averaging the pain grades across these three pain types, for each individual and per measurement occasion. Higher average pain grades indicate more intense and/or more widespread pain. In the rest of this paper, pain grade refers to this average pain grade.

**Table 1:** Operationalization of the chronic pain grade scale in the current study.

	Pain grade				
	0	1	2	3	4
Pain frequency [0–4]	[0]	[1–4]	[1–4]	[1–4]	[1–4]
Pain intensity [0–9]	–	[0–4]	[5–9]	[5–9]	[5–9]
Pain interference [0–6]	–	[0–2]	[0–2]	[3–4]	[5–6]

The pain grade measure can be taken to capture the degree of problematic pain, which is defined as having a high pain intensity associated with either significant distress or disability, or being experienced as multiple types of pain^39^. A pain grade of at least 2 on all three pain types can be taken to reflect generalized, problematic pain^39^.

#### Sleep

*Insomnia symptoms* were measured with the Insomnia Severity Index (ISI)^40^, from T1 to T4. The ISI consists of seven items regarding sleep problems during the last 6 months, each with ratings from 0 (“no problems at all”) to 4 (“very much”). Total scores range from 0 to 28. It has been used on a sample of Swedish youths with chronic pain^41^, and it has shown significant correlations with other measures of sleep quality, such as the Pittsburgh Sleep Quality Index, sleep diaries, and polysomnography^42^. A total score of 9 has been suggested as an appropriate cut-off for insomnia in adolescents^43^. The internal consistency in the current sample was α = 0.85 for T1, α = 0.84 for T2, α = 0.86 for T3, α = 0.87 for T4.

*Pre-sleep cognitive-emotional arousal* and *Pre-sleep behavior* were measured from T1 to T4 by using two subscales of the Adolescent Sleep Hygiene Scale, revised version (ASHS-R)^44^. ASHS is a questionnaire including 28 items with possible responses on Likert scales ranging from 0 (“Always”) to 5 (“Never”). The 6-item *cognitive-emotional sub-scale* [0–30] measures pre-sleep cognitive-emotional arousal (PSCEA) and includes items assessing worrying or ruminating in bed or feeling strong emotions in proximity to bedtime. The 3-item *behavioral arousal subscale* [0–15] measures pre-sleep behavior (PSB) and includes items assessing behavior before going to bed, or in bed, that increase physiological arousal and keeps you awake, such as talking on the phone or playing video games (3 items). The internal consistency in the current sample for the cognitive-emotional subscale was: 0.83 for T1, 0.83 for T2, 0.85 for T3, and 0.85 for T4; for the behavioral arousal subscale, the internal consistency was: 0.63 for T1 (mean inter-item correlation = 0.361), 0.67 for T2 (mean inter-item correlation = 0.403), 0.67 for T3 (mean inter-item correlation = 0.414), and 0.65 for T4 (mean inter-item correlation = 0.512). To represent change, we calculated the difference between pre-sleep behavior at T1 and T4 (T4-value minus T1-value) and used it as a predictor of the trajectories of pain grade and insomnia symptoms.

*Sleep duration* was measured by using the School Sleep Habits Survey for adolescents (SSHS)^45^, where the respondent is asked to estimate when they usually went to bed, how long time they usually took to fall asleep, and at what time they usually woke up, on average weekdays and average weekend days, during the last two weeks. In the current study, we included the average sleep time on weekdays and the average sleep time on weekends separately. Sleep duration was measured by calculating the time from bedtime to wake time, subtracting the reported time it took to fall asleep.

*Sleep phase* was also assessed with the SSHS^45^, and operationalized as hours and minutes between midnight and sleep midpoint (the mid-point between sleep onset and wake time) on weekend days^46^. However, most adolescents’ biological clocks are out of sync with school times, resulting in a general lack of sleep and accumulated sleep debt during weekdays for which they then compensate by sleeping in on weekends^47^. To adjust sleep phase for this confounder *sleep debt*, we utilized the correction that is used in the Munich Chronotype Questionnaire: In individuals who sleep longer on weekends than on weekdays, half the difference of sleep time during weekdays and sleep time during weekends is subtracted from the sleep phase-value^46^.

*Shift in sleep phase* was operationalized as change in adjusted sleep phase between T1 and T4 (T4-value minus T1-value), measured in minutes.

### Statistical analyses

Management of the raw data was handled in SPSS, version 24, and all subsequent analyses were conducted in Mplus, version 8.1. We applied multidimensional growth mixture modelling (GMM) following previously proposed guidelines^47–49^. GMM is a person-oriented analysis that extends latent growth curve modelling by incorporating a latent class variable that accounts for between-individual heterogeneity^48^. It assigns individuals to latent classes based on similar patterns of growth curves. A probability distribution decides what class the individual is most likely to belong to^49^.

To assess model fit, we put substantive value on meaningful, theoretical interpretability of the class solution^47,48^. We also used two types of statistical indices: the sample size-adjusted Bayesian Information Criteria (SSA-BIC) and the adjusted Lo-Mendell-Rubin likelihood ratio test (LMR-LRT)^48^. In addition, we considered entropy, as opted for the model with the least restrictions on model parameters, while also encountering the least convergence problems^50^: First, separate growth models were constructed on insomnia symptoms and pain grade. Low model fit and significant variance factors indicate heterogeneity in growth trajectories^48^. Secondly, a multidimensional growth mixture model was constructed on these two growth models simultaneously. We opted for an explorative approach^49^ and fitted latent class growth analyses (LCGA) with 2 to 7 classes, to establish the optimal number of classes. Age and gender were included as covariates in the analysis. We then utilized Wald χ^2^-tests to compare a zero-variance model (LCGA) to a model with class-invariant variances (GMM-CI), as well as to explore whether equal variances across the classes fitted the data as well as class-varying variances (GMM-CV). As a general rule, the preferred model is as freely estimated as possible while not encountering convergence problems^48^. Convergence problems indicate that the model is inadequate, that it may be overparameterized, and that a more parsimonious model is preferred^49^. Thereafter, we successively freed parameters—first class-invariant parameters, then class-varying parameters, until the optimal model was established. Since freeing parameters may change what number of classes is optimal^48^, we continuously tested 2 to 7 classes for both the class-invariant, and the class-varying models.

For incorporating the predictors (sleep phase, pre-sleep cognitive-emotional arousal and pre-sleep behavior, as well as change scores of those measures) into the GMM, we utilized the “three-step approach”, consisting of: (1) estimating an unconditional GMM with covariates included, (2) assigning individuals to latent classes while accounting for uncertainty of class membership, and (3) including predictors of the class-variable and within-class growth factors. Benefits of this approach is that the parameters of the mixture model are not influenced by the auxiliary predictor variable, while still accounting for uncertainty rate in class membership^47^. Three sets of analyses were done: First, differences among the classes on selected baseline variables were compared via Wald χ^2^-tests. Secondly, a multinomial regression analysis was done to assess if baseline sleep phase, pre-sleep cognitive-emotional arousal and pre-sleep behavior could predict class membership. Third, we examined the effect of sleep-related predictors on within-class growth factors (intercept and slope) of insomnia symptoms and pain grade. More information on statistical indices and criteria can be found in the Supplementary Information.

For handling missing data, we used full information maximum likelihood estimation (FIML)^51^.

## Results

### Estimation of the best fitting multidimensional growth mixture model

First, we established that latent basis growth curves fitted the data best. Second, we opted for a CI-GMM as the best-fitting model, since it fitted the data better than the LCGA while not encountering convergence problems (which the GMM-CV did), and it yielded theoretically meaningful classes that facilitated interpretation. Third, we found that a four-class solution of a GMM with class-invariant variances fitted the data best, when comparing theoretical meaningfulness and model fit indices of 2 to 7 classes. Details on model fit indices are described in Table 2. For more detailed information on these analyses, see the Supplementary information.

**Table 2:** Comparison of growth mixture models on insomnia symptoms and pain grade, on selected fit statistics (total N = 2755).

Fit statistics	2 Classes	3 Classes	4 Classes	5 Classes	6 Classes	7 Classes
**LCGA**						
LL (no. of parameters)	− 71,665.31	− 71,405.11	− **71,168.38**	− 71,051.37	− 70,993.83	− 70,857.36
SSABIC	143,477.68	142,980.99	**142,531.26**	14,230.96	142,109.60	141,980.37
Entropy	0.796	0.754	**0.778**	0.785	0.757	0.764
Adj. LMR-LRT (*p*)	2489.90 (< 0.001)	507.59 (0.027)	**461.79 (0.014)**	228.26 (0.201)	229.29 (0.108)	149.18 (0.375)
Class sizes in %	73.6–26.4	60.8–9.9	**58.1–8.1**	58.8–7.4	51.8–5.0	50.0–3.0
**GMM-CI**						
LL (no. of parameters)	− 71,074.35	− 70,970.12	− **70,884.83**	− *70,817.40*	− *70,755.91*	− *70,707.20*
SSABIC	142,343.20	142,158.46	**142,011.60**	*141,900.46*	*141,801.19*	*141,727.49*
Entropy	0.850	0.776	**0.786**	*0.794*	*0.778*	*0.786*
Adj. LMR-LRT (*p*)	600.68 (< 0.001)	203.32 (< 0.001)	**166.38 (0.048)**	*131.54 (0.059)*	*119.96 (0.690)*	*101.53 (0.173)*
Class sizes in %	84.3–15.7	74.0–11.7	**68.7–4.9**	*65.0–2.9*	*61.0–3.0*	*60.2–1.3*
**GMM-CV**^**a**^						
LL (no. of parameters)	− 70,529.12	− 70,673.47	**− 70,567.67**	− 70,502.04	*− 70,421.09*	*− 70,378.90*
SSABIC	141,266.97	141,598.37	**141,438.95**	141,359.86	*141,250.16*	*141,217.96*
Entropy	0.682	0.657	**0.683**	0.687	*0.719*	*0.742*
Adj. LMR-LRT (*p*)	1679.80 (< 0.001)	579.38 (< 0.001)	**174.62 (0.067)**	129.79 (0.751)	*141.73 (0.409)*	*83.42 (0.158)*
Class sizes in %	53.2–46.8	50.5–14.6	**48.9–7.6**	48.6–7.6	*46.8–2.6*	*45.7–1.4*

The columns in bold text represent the optimal class number. The columns in italics represent models that encountered convergence problems.

*LCGA* latent class growth analysis, *GMM-CI* growth mixture model with class-invariant variances and covariances, *GMM-CV* growth mixture model with class-varying variances and covariances. Columns in italics represent inadmissible solutions. Columns in bold represent the optimal solution, *LL* loglikelihood, *SSABIC* sample size adjusted Bayesian information criteria, *Adj. LMR-LRT* adjusted Lo-Mendell-Rubin likelihood ratio test.

^a^For the class-varying models to properly converge, the variance of the intercept factors had to be constrained to be equal across classes, and the slope variances in the largest class had to be constrained to zero.

### Class characteristics

#### Class descriptions

**Figure 1:**
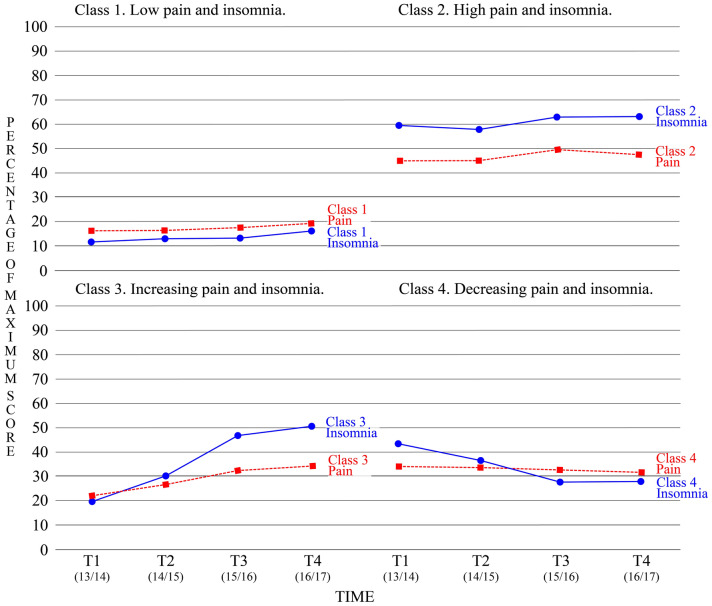
Longitudinal class solution of conjoint pain and insomnia development. The trajectories of pain are in red color (dotted line), and the trajectories of insomnia are in blue (solid line). The y-axis in the diagrams, which ranges from 0 to 100, represents percentage of maximum score of both insomnia symptoms and pain grade to facilitate comparisons of the trajectories. The x-axis represents measurement occasion, ranging from T1 to T4, and the numbers within parentheses are the age cohorts (in years) at each time point.

Figure 1 shows the conjoint trajectories of pain grade and insomnia symptoms of the four classes. Class 1 (n = 1893; 68.7% of total sample) was primarily characterized by consistently low levels of pain grade and insomnia, although both trajectories increased slightly. This class was labeled “Low pain and insomnia”. Class 2 (n = 134; 4.9%) was characterized by high levels of pain grade and insomnia symptoms throughout the measured period, although the trajectory of insomnia symptoms increased slightly. This class was labeled “High pain and insomnia”. Class 3 (n = 383; 13.9%) was characterized by increasing trajectories of both pain grade and insomnia symptoms. We labeled this class “Increasing pain and insomnia”. Class 4 (n = 345; 12.5%) was characterized by decreasing trajectories of pain grade and insomnia symptoms, although the decrease in pain grade was modest. This class was labeled “Decreasing pain and insomnia”.

#### Baseline characteristics of classes

Table 3 compares the classes on baseline variables. All variables, except for low SES and sleep phase shift, differed depending on class. Class 2 (high pain and insomnia) consistently reported a more severe profile than the other classes. About two thirds (68.7%) of the class-members were girls. This class reported the highest levels of pre-sleep cognitive-emotional arousal and pre-sleep behavior. About half of the class-members fulfilled the criteria for clinical anxiety and clinical depression. Furthermore, class 2 reported the shortest average sleep times and the latest sleep phase. Compared to class 1 (low pain and insomnia), class 2 reported a more than 13-fold prevalence of generalized problematic pain. Class 3 (increasing pain and insomnia) generally showed low symptoms at baseline, but reported a large shift towards a later sleep phase and the most profound deterioration of pre-sleep cognitive-emotional arousal. Class 4 (decreasing pain and insomnia) showed the second highest symptom levels on most variables at baseline, but also the smallest shift in sleep phase and was the only class to show an improvement in pre-sleep cognitive-emotional arousal.

**Table 3:** Sociodemographic data (mean or percentage) and differences of classes at baseline (T1).

	Class 1 (n = 1893)	Class 2 (n = 134)	Class 3 (n = 383)	Class 4 (n = 345)	Total (n = 2755)	Wald *χ*^2^ (p < 0.05)
Class probability	0.919	0.860	0.805	0.770		*–*
**Demographic variables**						
Age	13.63^a^	13.76^bc^	13.68^abd^	13.73^cd^	13.66	11.93*
Gender, proportion girls	41.8%	68.7%^a^	54.8%	63.1%^a^	47.6%	95.97*
Immigrant background	28.4%^ab^	40.3%^c^	30.0%^ad^	32.3%^bcd^	29.7%	8.91*
Low SES	9.2%	14.2%	9.4%	11.8%	9.8%	4.29
Clinical anxiety	5.5%	45.1%	13.9%	31.4%	11.9%	184.66*
Clinical depression	6.4%	50.0%	18.5%	32.9%	13.6%	218.21*
Stress	16.81	41.73	25.25	34.89	21.40	415.6*
**Pain variables**						
Average pain grade [0–4]	0.64	1.87	0.91	1.54	0.85	396.40*
Generalized problematic pain	1.7%^a^	22.4%	2.9%^a^	11.8%	4.1%	63.98*
Musculoskeletal pain frequency [0–4]	0.67	2.16	1.02	2.16	0.91	185.91*
Headache frequency [0–4]	0.66	2.12	0.92	1.77	0.91	191.85*
Abdominal pain frequency [0–4]	0.49	1.71^a^	0.84	1.63^a^	0.77	190.68*
Musculoskeletal pain intensity [0–9]	1.34	4.06	2.25	3.34	1.86	189.72*
Headache intensity [0–9]	1.92	4.75	2.47	4.75	2.42	363.23*
Abdominal pain intensity [0–9]	1.52	4.23	2.25	3.59	2.02	247.14*
Musculoskeletal pain interference [0–6]	0.39^ac^	1.79^b^	0.82^c^	1.79^ab^	0.73	32.53*
Headache interference [0–6]	0.55	2.09^a^	0.87	2.43^a^	0.95	355.07*
Abdominal pain interference [0–6]	0.40	1.88^a^	0.80	2.14^a^	0.79	253.51*
**Sleep variables**						
Insomnia symptoms [0–28]	3.32	17.26	5.91	12.74	5.57	4356.94*
Sleep duration week (hours:minutes)	8:15	6:39	7:48	7:16	7:59	269.75*
Sleep duration weekend (hours:minutes)	9:37	8:39^a^	9:32	9:16^a^	9:31	32.52*
Sleep phase (hours: minutes)	4:39	5:45	5:00^a^	5:10^a^	4:49	84.82*
Sleep phase change (hours:minutes)	00:24	− 00:03	00:23	00:08	00:21	6.45
PSCEA [0–30]	6.31	16.22	9.37	13.27	8.1	516.71*
PSCEA change	2.00^a^	0.88^ab^	5.17	− 0.54^b^	2.20	57.5*
PSB [0–15]	7.62	9.75	8.80^a^	9.42^a^	8.12	115.08*
PSB change	2.84^a^	1.13^b^	2.61^a^	1.48^b^	2.59	22.22*

The variables with the post-script “change” refer to the change score of the variables value at T4 minus the value at T1. These were included to highlight how these variables changed from T1 to T4. Pairwise comparisons, based on Wald *χ*^2^ tests, were done between all classes on all variables. Since most comparisons were significant, the superscripts represent specifically the class comparisons that did not significantly differ; all class comparisons without superscript differed at p < 0.05 level. The values with the same superscript (a, b, c or d) did not differ significantly from each other. For instance, regarding gender, only class 2 and 4 did not significantly differ from each other. Note that pairwise comparisons were not done on variables with non-significant overall tests.

*PSCEA* pre-sleep cognitive-emotional arousal, *PSB* pre-sleep behavior.

Significant values are in bold.

*p < 0.05.

### Sleep-related predictors of longitudinal trajectories and change

#### Sleep phase and pre-sleep cognitive-emotional arousal predict diverging trajectories of pain grade and insomnia symptoms

To explore whether sleep phase, pre-sleep cognitive-emotional arousal and pre-sleep behavior at baseline could predict diverging concurrent trajectories of pain grade and insomnia symptoms, we conducted a multinomial regression analysis and focused on comparing the “Low pain and insomnia” class (1) with the “Increasing pain and insomnia” class (2), and the “High pain and insomnia” class (3) with the “Decreasing pain and insomnia” class (4). Note that all classes were compared, but that for clarity only the above-mentioned comparisons are presented here. See the Supplementary Information for the full set of analyses. As can be seen in Table 4, a later sleep phase and higher ratings on pre-sleep cognitive-emotional arousal (but not pre-sleep behavior) at baseline predicted increased insomnia symptoms and pain grade. An earlier sleep phase and less pre-sleep cognitive-emotional arousal at baseline predicted reduced pain grade and insomnia symptoms.

**Table 4:** The influence of sleep phase and pre-sleep cognitive-emotional arousal and behavior on class membership, using multinomial logit regressions.

	Low (Class 1) versus increasing (Class 3)	High (Class 2) versus decreasing (Class 4)
Sleep phase	0.003*	− 0.004*
PSCEA	0.101*	− 0.062*
PSB	0.016^ns^	− 0.001^ns^

Displayed are the predictors’ effects on the increasing Class 3, with the low Class 1 as reference, and the decreasing Class 4, with the high Class 2 as reference.

Unstandardized coefficients are shown. The predictors are measured at baseline (T1).

^*ns*^ non-significant at *p* < 0.05 level, *PSCEA* pre-sleep cognitive-emotional arousal, *PSB* pre-sleep behavior.

*p < 0.05*.*

Significant values are in bold.

#### Sleep phase, pre-sleep cognitive-emotional arousal and pre-sleep behavior predict within-class change of insomnia symptoms and pain grade

To assess whether sleep phase, pre-sleep cognitive-emotional arousal and pre-sleep behavior could predict initial levels and longitudinal change in pain grade and insomnia symptoms, we regressed the intercepts and slopes of pain grade and insomnia symptoms on the predictors at baseline, as well as on their change scores, in a multivariate regression analysis. Table 5 depicts the results of these analyses. More pre-sleep cognitive-emotional arousal predicted higher levels of both pain grade and insomnia symptoms, a later sleep phase predicted more insomnia symptoms, and higher levels of pre-sleep behavior predicted more insomnia symptoms as well as a slighter increase or a decrease in insomnia symptoms. A greater shift to a later sleep phase and a larger increase in reported pre-sleep cognitive-emotional arousal predicted a steeper longitudinal increase in insomnia symptoms, whereas only a greater increase of pre-sleep cognitive-emotional arousal predicted a steeper longitudinal increase of pain grade.

**Table 5:** The predictive effects of sleep-related factors on within-class change (intercept and slope factors) in pain grade and insomnia symptoms.

	Insomnia intercept (*p*)	Insomnia slope (*p*)	Pain intercept (*p*)	Pain slope (*p*)
Sleep phase at baseline	**0.005 (0.051)**	0.001 (0.436)	0.003 (0.527)	− 0.002 (0.588)
Sleep phase change	–	**0.003 (0.021)**	–	− 0.003 (0.289)
PSCEA at baseline	**0.496 (< 0.001)**	0.043 (0.098)	**1.006 (< 0.001)**	0.093 (0.054)
PSCEA change	–	**0.216 (< 0.001)**	–	**0.485 (< 0.001)**
PSB at baseline	**0.125 (0.015)**	**− 0.076 (0.033)**	0.101 (0.335)	− 0.125 (0.058)
PSB change	–	− 0.019 (0.531)	–	0.018 (0.764)

Coefficients in bold are significant at *p* < 0.05 level. “Change” refers to a change score, where the value at T1 is subtracted from the value at T4.

*PSCEA* pre-sleep cognitive-emotional arousal-causing, *PSB* pre-sleep behavior.

## Discussion

The current study examined the co-development of pain and insomnia throughout adolescence. We identified four subgroups with different concurrent developmental trajectories of pain and insomnia symptoms. The results suggest that adolescents with consistently high pain and insomnia may constitute a unique subgroup that is particularly vulnerable. Furthermore, the study established that both a late sleep phase and pre-sleep cognitive-emotional arousal (but not pre-sleep behavior) predicted pain and insomnia symptoms.

The current results show that trajectories of pain and insomnia co-occur to a high degree in all subgroups, regardless of the direction of the trajectories. The relationship between pain and insomnia is in line with previous work^2,3,52^. Given the substantial variability in the trajectories, however, there may be adolescents with high symptoms levels of either pain or insomnia included. This finding also indicates that lack of such relationship in previous study samples may be due to a mix of subgroups with both increasing and decreasing trajectories, resulting in a null result when looking at grand means. We are not aware of any previous study that has explored individual differences in longitudinal trajectories of co-developmental pain and insomnia. Previous studies have primarily focused on associations between variable means, for example that mean levels of insomnia symptoms at one timepoint can predict mean levels of pain at a later timepoint. Such an approach fails to capture continuity of trajectories over time and cannot demonstrate that pain and insomnia continuously co-change within individuals.

The four classes identified in the current study correspond to previous studies on longitudinal pain trajectories in adolescents. Most commonly, four or five classes have been identified as the optimal solution in studies on pain, typically including one class with persistent low pain, one with persistent high pain, as well as two or three classes of fluctuating, increasing or decreasing pain^53^. Few studies have included insomnia trajectories in adolescents, but one study identified trajectories similar to those found in regard to pain^54^. In the current study, the yearly measurements and the 6-month coverage of the measures likely means that the trajectories primarily represent stable aspects of pain and insomnia. Future work with more frequent testing, more transient measures or longer measurement periods could reveal long-term trajectories of change that we are unable to show with the current study design. Such study designs could possibly help characterizing dynamic and complementary trajectories, as well as identifying more state-like aspects of pain and insomnia. Most of the adolescents in the current sample had consistently low pain and insomnia levels throughout the measured period (Class 1, 68.7%), which is to be expected in a sample derived from the general population. About 14 percent of the sample (Class 3, 13.9%) were characterized by increasing levels of both pain and insomnia symptoms. This subgroup showed the largest shift towards a later sleep phase and the largest increase in pre-sleep cognitive-emotional arousal. A third subgroup showed decreasing levels of pain and insomnia symptoms (Class 4, 12.5%). Earlier sleep phases and less pre-sleep cognitive-emotional arousal could predict these decreasing trajectories, compared to the high stable trajectories. It may be that early, proactive strategies that focus on late sleep phases and pre-sleep cognitive-emotional arousal help in preventing the concurrent development of pain and insomnia. This may be further investigated in experimental studies that manipulate sleep–wake patterns and pre-sleep habits directly in order to demonstrate their causal effect on pain and insomnia.

A subgroup of consistently high pain and insomnia symptoms was identified (Class 2.4, 9%), constituting about 5 percent of the overall sample. Girls made up two thirds of this subgroup. Adolescents in this subgroup showed higher levels of symptoms on most of the baseline characteristics examined, including stress, depression, and anxiety. They also reported the shortest sleep times, the latest average sleep phase, as well as the most problematic pre-sleep behavior. Compared to Class 1 (low pain and insomnia), they reported a more than 13-fold rate of generalized problematic pain. This indicates that the subpopulation of adolescents with comorbid pain and insomnia is associated with a particularly complex illness-profile, and indeed may benefit from specialized interventions. However, future studies should thoroughly explore potential differences between comorbid pain and insomnia and subgroups of adolescents with only pain or insomnia problems.

Pre-sleep cognitive-emotional arousal was the most consistent predictor of change in both pain and insomnia symptoms. This is in line with previous research suggesting that cognitive-emotional arousal may delay sleep onset and lower sleep quality^24^, and that worry, rumination and emotional distress are associated with the sleep-pain relationship^3,55^. Additionally, stress responses or arousal has been proposed to explain the sleep-pain relationship^5^, as well as influencing melatonin secretion^56^. Pre-sleep arousal may have both a moderating and a mediating effect on comorbid pain and insomnia. We encourage future studies to explore the efficacy of hybrid interventions for comorbid pain and insomnia that incorporate a focus on pre-sleep cognitive-emotional arousal. Notably, sleep hygiene practices (including pre-sleep behavior) are often a main focus in insomnia-interventions for adolescents^7^.

Pre-sleep behavior did not consistently predict conjoint trajectories of pain and insomnia. However, pre-sleep behavior and somatic pre-sleep arousal have previously been associated with insomnia in adolescents suffering from chronic pain^24^. Plausible explanations for our null finding that require further investigation lie either in that this finding is specific to conjoint pain and insomnia symptoms, that the internal consistency of the behavioral arousal subscale was insufficient in the current study, or that the items in this subscale are relatively positive in their valence.

The association between sleep phase and trajectories of pain and insomnia symptoms may help explain inconsistent results of non-pharmacological treatments for comorbid pain and insomnia^57,58^. Social and behavioral factors related to adolescent sleep–wake patterns, which are not a target in psychological treatments, may impede or confound treatment effects. In addition, pharmacological, social or psychological interventions may be effective in treating the conjoint development of pain and insomnia, including melatonin^7^, synchronizing adolescents’ school time with their biological clock^59^, and CBT^60^. The current results may indicate that the effect of melatonin on pain is mediated through insomnia symptoms, practically meaning that advancing adolescents’ sleep phase may lead to a reduction in insomnia symptoms, which, in turn, may reduce pain^56^.

A strength of the current study is the large population sample of adolescents followed over four yearly measurement occasions. This large set of data allowed us to use class-invariant GMM and account for potential sub-distributions via the class-variable, as well as between-individual differences and within-individual temporal change via the growth factors. Accounting for within-class variance often decreases the risk of identifying spurious classes^48^ and reduces the risk of bias in the trajectories^61^. The use of latent basis and unspecified growth curves is also a strength of the study, since it does not impose any restrictions to the shape of longitudinal change^32^.

Some limitations need to be considered. First, for current purposes, we summarized the experience of three common types of pain (musculoskeletal pain, headache and abdominal pain) in an average pain grade, taking into account the intensity, frequency, and interference of the experience. Although this composite measure captures covariance across different pain types with insomnia^5^, future studies might want to focus on co-developmental patterns in specific pain types, which would also allow interpretation of the pain grades in terms of pain intensity and pain-related disability only^14,62^. Indeed, the co-development of pain and insomnia might turn out to be different for specific pain types^63^. A second limitation is that only self-report measures of complex constructs were used. Since objective and subjective sleep measures appear to partly measure different aspects of sleep^9^, future studies should include also objective sleep-related measures. While the ISI has been used in a few studies in adolescents^43,64,65^, it has not been extensively validated in this population. A third limitation is that diverging trajectories were not perfectly overlapping at baseline, which may (although unlikely) have confounded the multinomial analysis of diverging trajectories.

In conclusion, the current study brings novel insights into how pain and insomnia symptoms longitudinally co-develop in adolescents. We identified four unique subgroups, showing that longitudinal trajectories of pain and insomnia closely follow each other, and that sleep phase and pre-sleep cognitive-emotional arousal and behavior could predict the trajectories. A considerable portion of adolescents have either high persistent or increasing levels of problematic pain and insomnia. Adolescence constitute a melting pot of factors contributing to the development of both insomnia and pain^5^, and therefore appears to be an important developmental stage for preventing these conditions from becoming chronic sources of disability and suffering. A sleep-phase focus may be relevant in interventions for comorbid pain and insomnia in adolescents, and pre-sleep cognitive-emotional arousal seems to be of particular importance to focus on in psychological interventions.

## Supplementary Information

Supplementary Information.

## Data Availability

The syntax code and the variance–covariance matrix that the study’s analyses are based on are provided in the Supplementary information, which allows for replication of the analyses. The raw dataset may be made available after permission from the Three Cities Study PI Katja Boersma (katja.boersma@oru.se) upon request.
