# Supplementary material for: Sleep phase and pre-sleep arousal predicted co-developmental trajectories of pain and insomnia within adolescence
Source: Sci Rep. 2022 Mar 16;12:4480. doi: 10.1038/s41598-022-08207-y (PMC8927379; doi:10.1038/s41598-022-08207-y)
Supplement: Supplementary file 1 — Supplementary Information. [file 41598_2022_8207_MOESM1_ESM.pdf]

Sleep phase and pre-sleep arousal predicted co-developmental trajectories of pain and insomnia within adolescence.

*Tor Arnison[1], Martien G. S. Schrooten[1], Serena Bauducco[1], Markus Jansson-Fröjmark[2,3], and Jonas Persson[1,4].

[1]School of Law, Psychology and Social Work, Örebro University, Örebro, Sweden.

[2]Centre for Psychiatry Research, Department of Clinical Neuroscience, Karolinska Institute, Stockholm, Sweden.

[3]Stockholm Health Care Services, Region Stockholm.

[4]Aging Research Center (ARC), Karolinska Institute and Stockholm University, Stockholm, Sweden.

**Supplemental Information**

**7.1. Test of measurement invariance of the pain intensity measures.**

To explore whether the pain construct remained stable across time and if the deviation of the measures of abdominal pain intensity and headache intensity at timepoint 3 (for the last 2 months instead of for the last 6 months) had an effect on the measures, we tested each pain type for measurement invariance. To accomplish this, we first constructed latent variables of each pain type, where pain intensity, pain frequency and pain interference represented the manifest indicators for each pain type at each timepoint. These were subsequently incorporated into three longitudinal confirmatory factor analyses, and tested for configural invariance, metric/weak invariance and scalar/strong invariance. As can be seen in **Table S1** below, all pain types held for the assumption of strong measurement invariance, which indicates that the constructs remained stable across all timepoints and that the deviating time scaling at timepoint 3 did not have an impact on the constructs.

**Table S1**
Model fit statistics for the tests of invariance in the three longitudinal confirmatory factor analyses of musculoskeletal pain, headache and abdominal pain.

| Model tested | $\chi^2$ | *Df* | *P* | RMSEA | RMSEA 90% CI | CFI | ΔCFI |
|---|---|---|---|---|---|---|---|
| | | | | Model estimates | | | |
| *Musculoskeletal pain* | | | | | | | |
| Null model | 17226.06 | 66 | <.001 | --- | --- | --- | --- |
| Configural invariance | 162.99 | 33 | <.001 | .038 | .032 - .044 | .992 | --- |
| Metric (weak) invariance | 169.20 | 39 | <.001 | .035 | .030 - .040 | .992 | .000 |
| Scalar (strong) invariance | 216.53 | 45 | <.001 | .037 | .032 - .042 | .990 | .002 |
| *Headache* | | | | | | | |
| Null model | 15734.21 | 66 | <.001 | --- | --- | --- | --- |
| Configural invariance | 187.33 | 33 | <.001 | .041 | .036 - .047 | .990 | --- |
| Metric (weak) invariance | 199.76 | 39 | <.001 | .039 | .033 - .044 | .990 | .000 |
| Scalar (strong) invariance | 216.96 | 45 | <.001 | .037 | .032 - .042 | .989 | .001 |
| *Abdominal pain* | | | | | | | |
| Null model | 14533.26 | 66 | <.001 | --- | --- | --- | --- |
| Configural invariance | 205.66 | 33 | <.001 | .044 | .038 - .049 | .988 | --- |
| Metric (weak) invariance | 231.28 | 39 | <.001 | .042 | .037 - .048 | .987 | .001 |
| Scalar (strong) invariance | 246.81 | 45 | <.001 | .040 | .036 - .045 | .986 | .001 |

Note. Each longitudinal confirmatory factor analysis consisted of latent variables of pain that, in turn, were constructed from pain intensity, pain frequency and pain interference as manifest indicators.

**7.2. Model building.**

First, linear growth curve models of insomnia symptoms and pain were constructed. They showed adequate fit to the data ($\chi^2(22) = 289.44$, $p = .066$, RMSEA = .066, CFI = .964) and significant variance in growth factors, which indicated unobserved heterogeneity in the growth curves that may be better explained via GMM. Adding quadratic growth factors was

met with convergence issues, and there were not sufficient degrees of freedom to properly specify the quadratic model. Specifying latent basis growth curves improved model fit compared to the linear model ($\Delta\chi^2(4) = 17.59$, $p = .001$, ΔRMSEA = .006, ΔCFI = .002). We therefore opted for basing the GMM on latent basis growth curves. Note that we also completed all analyses with linear growth curves, and they showed comparable results as with the latent basis growth curves.

Secondly, we explored the optimal class number in LCGA's, and found the 4-class solution to be optimal. We then explored which level of GMM would fit the data best, via Wald $\chi^2$-tests. A model with class-invariant variances (GMM-CI) fitted the data significantly better than a zero-variance model (LCGA; Wald $\chi^2(8) = 280.22$, $p < .001$). When estimating a GMM with class-varying variances (GMM-CV), the intercept variances had to be constrained to be equal across classes, and the slope variances in the largest class had to be constrained to zero. Freeing the remaining three slope variances to be class-varying, compared to class-invariant, significantly improved the model (Wald $\chi^2(13) = 28.01$, $p = .009$). Note that this comparison did not take the plausibly negative effect of the zero-variance slope into account. Taken together, we opted for the more parsimonious GMM-CI as the best-fitting model, since it did not encounter convergence problems (which the GMM-CV did) and it yielded theoretically meaningful classes that facilitated interpretation. Third, we found that a four-class solution of a GMM with class-invariant variances fitted the data best, when comparing theoretical meaningfulness and model fit indices of 2 to 7 classes.

### 7.3. Longitudinal descriptive data of the key variables and sociodemographic data at T4

Longitudinal descriptive data of the key variables are depicted in **Table S2**. As can be seen, the values on the pain grades and insomnia symptoms increase in all classes except for in Class 4 ("Decreasing pain and insomnia"). This is to be expected, since both pain and insomnia increase across adolescence. Pre-sleep cognitive-emotional arousal and pre-sleep behaviors increased across the four measurement occasions in the general sample, and the average sleep phaseshifted to be 19 minutes later from timepoint 1 to timepoint 4.

**Table S2**
Descriptive statistics for key variables at T1 to T4.

| | | | Timepoint | | |
|---|---|---|---|---|---|
| | | 1 | 2 | 3 | 4 |
| Pain grade | | | | | |
| Class 1 | [0-100] | 15.96 | 16.20 | 17.23 | 18.92 |
| | [0-4] | 0.64 | 0.65 | 0.69 | 0.76 |
| Class 2 | [0-100] | 45.32 | 45.47 | 49.33 | 45.73 |
| | [0-4] | 1.81 | 1.82 | 1.97 | 1.83 |
| Class 3 | [0-100] | 23.24 | 28.01 | 33.83 | 36.18 |
| | [0-4] | 0.93 | 1.12 | 1.35 | 1.45 |
| Class 4 | [0-100] | 36.57 | 34.12 | 33.80 | 33.19 |
| | [0-4] | 1.46 | 1.36 | 1.35 | 1.33 |
| Insomnia symptoms | | | | | |

| | | | | | |
|---|---|---|---|---|---|
| Class 1 | [0-100] | 11.81 | 12.93 | 14.29 | 16.47 |
| | [0-28] | 3.31 | 3.62 | 4.00 | 4.61 |
| Class 2 | [0-100] | 59.46 | 57.70 | 63.27 | 63.14 |
| | [0-28] | 16.65 | 16.16 | 17.72 | 17.68 |
| Class 3 | [0-100] | 20.80 | 31.27 | 47.54 | 51.29 |
| | [0-28] | 5.82 | 8.76 | 13.31 | 14.36 |
| Class 4 | [0-100] | 44.54 | 36.36 | 29.07 | 29.37 |
| | [0-28] | 12.47 | 10.18 | 8.14 | 8.22 |
| Sleep phase [hours:minutes] | | 4:51 | 4:59 | 5:06 | 5:10 |
| PSCEA [0-30] | | 8.14 | 8.95 | 9.75 | 10.19 |
| PSB [0-15] | | 8.11 | 9.06 | 10.00 | 10.54 |

Note: All the means and standard deviations were estimated using full information maximum likelihood estimation (FIML). Pain grade and insomnia symptoms are presented in percentage of maximum score, the average score within each of the four classes. Regarding Sleep phase, pre-sleep cognitive-emotional arousalCEA and pre-sleep behaviors, the averages in the overall samples are presented.
PSCEA = Pre-sleep cognitive-emotional arousal.
PSB = Pre-sleep behaviors.

Descriptive data on sociodemographic variables are detailed in Table S3 below:

Table S3. Sociodemographic data (mean or percentage) and differences of classes at T4

| | Class 1 (n=1893) | Class 2 (n=134) | Class 3 (n=383) | Class 4 (n=345) |
|---|---|---|---|---|
| Class probability | 0.919 | 0.860 | 0.805 | 0.770 |
| *Demographic variables* | | | | |
| Age | 16.77 | 16.82 | 16.85 | 16.96 |
| Low SES | 5.5 % | 16.4 % | 8.9 % | 6.6 % |
| Clinical anxiety | 11.6 % | 61.8% | 48.1 % | 29.1 % |
| Clinical depression | 8.7 % | 29.1 % | 35.0 % | 18.7 % |
| Stress | 20.55 | 43.84 | 36.94 | 30.50 |
| *Pain variables* | | | | |
| Average pain grade [0-4] | 0.75 | 1.86 | 1.49 | 1.37 |
| Generalized problematic pain | 2.7 % | 12.0 % | 10.4 % | 5.6 % |
| Musculoskeletal pain frequency [0-4] | 0.92 | 2.34 | 1.76 | 1.61 |
| Headache frequency [0-4] | 0.81 | 2.02 | 1.64 | 1.52 |
| Abdominal pain frequency [0-4] | 0.65 | 2.02 | 1.64 | 1.52 |
| Musculoskeletal pain intensity [0-9] | 1.96 | 4.77 | 3.61 | 3.23 |
| Headache intensity [0-9] | 2.30 | 4.67 | 3.84 | 3.77 |
| Abdominal pain intensity [0-9] | 1.89 | 3.91 | 3.60 | 3.27 |
| Musculoskeletal pain interference [0-6] | 0.38 | 2.05 | 1.22 | 1.76 |
| Headache interference [0-6] | 0.57 | 2.20 | 1.65 | 2.13 |
| Abdominal pain interference [0-6] | 0.45 | 1.48 | 1.56 | 1.89 |

| *Sleep variables* | | | | |
|---|---|---|---|---|
| Insomnia symptoms [0-28] | 4.59 | 18.39 | 14.94 | 8.31 |
| Sleep duration week (hours:minutes) | 7:43 | 6:31 | 6:47 | 7:18 |
| Sleep duration weekend (hours:minutes) | 9:02 | 8:40 | 8:41 | 8:51 |
| Sleep phase (hours: minutes) | 4:59 | 5:24 | 5:19 | 5:21 |
| PSCEA [0-30] | 8.05 | 17.62 | 14.59 | 12.62 |
| PSB [0-15] | 10.24 | 10.67 | 11.40 | 11.33 |

PSCEA = Pre-sleep cognitive-emotional arousal.

PSB =Pre-sleep behaviors.

### 7.4. Multinomial regression analysis

The tables below details the full multinomial regression analysis examining if sleep phase, pre-sleep cognitive-emotional arousal and pre-sleep behaviors can predict class membership. **Table S4** has class 1 (low pain and insomnia) as reference class, and **Table S5** has class 2 (high pain and insomnia) as reference class.

**Table S4.** The influence of sleep phase, pre-sleep cognitive-emotional arousal and pre-sleep behaviors on class membership, using multinominal logit regressions. Class 1 (low pain and insomnia) is the reference class.

| | Class 1 versus Class 2 | Class 1 versus Class 3 | Class 1 versus Class 4 |
|---|---|---|---|
| Sleep phase | **0.007*** | **0.003*** | **0.003*** |
| PSCEA | **0.267*** | **0.101*** | **0.204*** |
| PSB | -0.011$^{ns}$ | 0.016$^{ns}$ | -0.012$^{ns}$ |

Note. Unstandardized coefficients are shown. The predictors are measured at baseline (T1). *$p$<.05.
$^{ns}$ = non-significant at $p$ < .05 level.
PSCEA = Pre-sleep cognitive-emotional arousal.
PSB = Pre-sleep behaviors.

**Table S5.** The influence of sleep phase, pre-sleep cognitive-emotional arousal and pre-sleep behaviors on class membership, using multinominal logit regressions. Class 2 (high pain and insomnia) is the reference class.

| | Class 2 versus Class 1 | Class 2 versus Class 3 | Class 2 versus Class 4 |
|---|---|---|---|
| Sleep phase | **-0.007*** | **-0.004*** | **-0.004*** |
| PSCEA | **-0.267*** | **-0.167*** | **-0.064*** |
| PSB | 0.011$^{ns}$ | 0.028$^{ns}$ | -0.001$^{ns}$ |

Note. Unstandardized coefficients are shown. The predictors are measured at baseline (T1).
*$p$<.05.
$^{ns}$ = non-significant at $p < .05$ level.
PSCEA = Pre-sleep cognitive-emotional arousal.
PSB = Pre-sleep behaviors.

7.5. Mplus Syntax.

7.5.1. Multidimensional GMM

```
Usevariables =
    a_cpg b_cpg c_cpg d_cpg
    a_cisi b_cisi c_cisi d_cisi;

Classes = c(4);

IDvariable = ID;

  DEFINE:

      ANALYSIS:
  type=mixture;

      MODEL:

 %overall%

i_cpg s_cpg | a_cpg@0 b_cpg@1 c_cpg* d_cpg*;
i_cisi s_cisi | a_cisi@0 b_cisi@1 c_cisi* d_cisi*;

a_cisi with b_cisi;
b_cisi with c_cisi;
c_cisi with d_cisi;
a_cpg with b_cpg;
b_cpg with c_cpg;
```

```
  c_cpg with d_cpg;
  a_cpg with a_cisi;
  b_cpg with b_cisi;
  c_cpg with c_cisi;
  d_cpg with d_cisi;

  i_cpg (m1);
  s_cpg (m2);

  i_cisi (m3);
  s_cisi (m4);

  i_cpg with s_cpg (c1);
  i_cisi with s_cisi (c2);
  i_cpg with i_cisi (c3);
  s_cpg with s_cisi (c4);
```

7.5.2. Baseline comparisons.

```
  Classes = c(4);

  IDvariable = ID;

  Nominal = N2;

Auxiliary = gender (dcat) dep (dcat) anx (dcat) ins (dcat) wspain2 (dcat) cult (dcat) ses (dcat);

Auxiliary = ses (du3step) stress (du3step) a_isi (du3step) a_msf (du3step) a_ce (du3step) a_ba
(du3step) a_pgmix (du3step) a_sjl (du3step) sovtid (du3step) wksov (du3step) wesov
(du3step) a_rint (du3step) a_hint (du3step) a_mint (du3step) a_rfrq (du3step) a_hfrq
(du3step) a_mfrq (du3step) a_rinf (du3step) a_hinf (du3step) a_minf (du3step)  a_msf
(du3step) age (du3step) wksov (du3step) wesov (du3step);

Auxiliary = dlt_msf (du3step) dlt_ce (du3step) dlt_ba (du3step);
```

```
ANALYSIS:

type = mixture;

MODEL:

%overall%

i_cpg s_cpg | a_cpg@0 b_cpg@1 c_cpg* d_cpg*;
i_cisi s_cisi | a_cisi@0 b_cisi@1 c_cisi* d_cisi*;

a_cisi with b_cisi;
b_cisi with c_cisi;
c_cisi with d_cisi;
a_cpg with b_cpg;
b_cpg with c_cpg;
c_cpg with d_cpg;
a_cpg with a_cisi;
b_cpg with b_cisi;
c_cpg with c_cisi;
d_cpg with d_cisi;

i_cpg;
s_cpg;
i_cisi;
s_cisi;
i_cpg with s_cpg;
i_cisi with s_cisi;
i_cpg with i_cisi;
s_cpg with s_cisi;

%C#1%
```

```
[N2#1@11.297];[N2#2@7.306];[N2#3@7.711];

%C#2%
[N2#1@1.145];[N2#2@2.903];[N2#3@0.254];

%C#3%
[N2#1@3.219];[N2#2@1.671];[N2#3@4.289];

%C#4%
[N2#1@-4.727];[N2#2@-1.542];[N2#3@-2.729];
```

7.5.3. Multinomial analysis.

```
Classes = c(4);

IDvariable = ID;

Nominal = N2;

Auxiliary = (r3step) a_ba a_ce a_msf ;

ANALYSIS:

type = mixture;

MODEL:

%overall%

%C#1%
  [N2#1@11.297];[N2#2@7.306];[N2#3@7.711];
```

```
%C#2%
[N2#1@1.145];[N2#2@2.903];[N2#3@0.254];

%C#3%
[N2#1@3.219];[N2#2@1.671];[N2#3@4.289];

%C#4%
[N2#1@-4.727];[N2#2@-1.542];[N2#3@-2.729];
```

7.8.3. Within-class analysis.

```
Classes = c(4);

IDvariable = ID;

Nominal = N2;

ANALYSIS:

type = mixture;

MODEL:

%overall%

i_cpg s_cpg | a_cpg@0 b_cpg@1 c_cpg* d_cpg*;
i_cisi s_cisi | a_cisi@0 b_cisi@1 c_cisi* d_cisi*;

a_cisi with b_cisi;
b_cisi with c_cisi;
c_cisi with d_cisi;
a_cpg with b_cpg;
```

```
b_cpg with c_cpg;
c_cpg with d_cpg;
a_cpg with a_cisi;
b_cpg with b_cisi;
c_cpg with c_cisi;
d_cpg with d_cisi;

i_cpg;
s_cpg;
i_cisi;
s_cisi;

i_cpg with s_cpg;
i_cisi with s_cisi;
i_cpg with i_cisi;
s_cpg with s_cisi;

a_msf;
a_ce;
a_ba;
dlt_msf;
dlt_ce;
dlt_ba;

s_cpg on a_msf a_ce a_ba;
s_cisi on a_msf a_ce a_ba;
i_cpg on a_msf a_ce a_ba;
i_cisi on a_msf a_ce a_ba;
s_cpg on dlt_msf dlt_ce dlt_ba;
s_cisi on dlt_msf dlt_ce dlt_ba;

a_msf with a_ce@0;
```

```
a_msf with a_ba@0;
a_ba with a_ce@0;
dlt_msf with a_msf@0;
dlt_msf with a_ce@0;
dlt_msf with a_ba@0;
dlt_ce with a_ce@0;
dlt_ce with a_ba@0;
dlt_ce with a_msf@0;
dlt_ba with a_ba@0;
dlt_ba with a_ce@0;
dlt_ba with a_msf@0;
dlt_msf with dlt_ba@0;
dlt_msf with dlt_ce@0;
dlt_ba with dlt_ce@0;

%C#1%
[N2#1@11.297];[N2#2@7.306];[N2#3@7.711];

%C#2%
[N2#1@1.145];[N2#2@2.903];[N2#3@0.254];

%C#3%
[N2#1@3.219];[N2#2@1.671];[N2#3@4.289];

%C#4%
[N2#1@-4.727];[N2#2@-1.542];[N2#3@-2.729];
```

7.6. Variance-covariance matrix.

```
Covariances
  A_MSF      A_CE       A_BA       A_CPG      B_CPG
  ________   ________   ________   ________   ________
```

| | | | | | |
|---|---|---|---|---|---|
| A_MSF | 7032.670 | | | | |
| A_CE | 78.707 | 38.055 | | | |
| A_BA | 73.021 | 10.084 | 14.422 | | |
| A_CPG | 209.423 | 57.932 | 16.297 | 468.923 | |
| B_CPG | 172.443 | 48.537 | 13.186 | 260.755 | 509.205 |
| C_CPG | 178.290 | 51.577 | 12.227 | 274.046 | 338.663 |
| D_CPG | 200.921 | 45.700 | 10.275 | 257.159 | 336.901 |
| A_CISI | 326.151 | 57.786 | 17.216 | 198.455 | 157.874 |
| B_CISI | 203.048 | 47.705 | 13.191 | 159.934 | 222.719 |
| C_CISI | 282.030 | 45.060 | 12.787 | 152.153 | 192.353 |
| D_CISI | 314.763 | 49.872 | 13.296 | 145.101 | 188.449 |
| DLT_MSF | -3983.841 | -44.183 | -33.283 | -128.441 | -113.930 |
| DLT_CE | -29.609 | -17.997 | -4.090 | -14.324 | 2.527 |
| DLT_BA | -51.042 | -6.175 | -9.082 | -7.288 | -4.702 |

Covariances

| | C_CPG | D_CPG | A_CISI | B_CISI | C_CISI |
|---|---|---|---|---|---|
| C_CPG | 574.235 | | | | |
| D_CPG | 401.351 | 591.845 | | | |
| A_CISI | 164.123 | 141.512 | 310.932 | | |
| B_CISI | 202.568 | 187.688 | 194.174 | 338.973 | |
| C_CISI | 266.789 | 228.827 | 190.598 | 239.031 | 406.940 |
| D_CISI | 236.535 | 280.140 | 180.850 | 229.167 | 290.335 |
| DLT_MSF | -65.150 | -120.506 | -145.750 | -24.746 | -76.417 |
| DLT_CE | 19.934 | 31.945 | -18.412 | 2.863 | 13.334 |
| DLT_BA | 0.463 | 3.969 | -10.393 | -3.565 | -5.855 |

Covariances

| | D_CISI | DLT_MSF | DLT_CE | DLT_BA |
|---|---|---|---|---|
| D_CISI | 456.276 | | | |

| | | | | |
|---|---|---|---|---|
| DLT_MSF | 1.695 | 7500.544 | | |
| DLT_CE | 29.741 | 56.421 | 40.858 | |
| DLT_BA | 0.765 | 52.193 | 8.758 | 17.035 |

References


[1] Jung T, Wickrama KAS. An introduction to latent class growth analysis and growth mixture modeling. Social and Personality Psychology Compass 2008;2:302-317.

[2] Morin AJS, Maïano C, Nagengast B, Marsh JM, Janosz M. General growth mixture analysis of adolescents' developmental trajectories of anxiety : the impact of untested invariance assumptions on substantive interpretations. Structural Equation Modeling: A Multidisciplinary Journal 2011;18:613-648.

[3] Nylund-Gibson K, Gruimm R, Quirk M, Furlong M. A latent transition mixture model using the three-step specification. Structural Equation Modeling: A Multidisciplinary Journal 2014;21:1-16.

[4] Sijbrandij JJ, Hoekstra T, Almansa J, Peeters M, Bultmann U, Reijneveld SA. Variance constraints strongly influenced model performance in growth mixture modeling: a simulation and empirical study. BMC Med Res Methodol 2020;20(1):276.

[5] Wickrama KAS, Lee TK, Walker O'Neal C, Lorenz FO. Higher-order growth curves and mixture modeling with Mplus: A practical guide. New York: Routledge, 2016.
